# Predictability of the dispersion of Fukushima-derived radionuclides and their homogenization in the atmosphere

**DOI:** 10.1038/srep19915

**Published:** 2016-01-28

**Authors:** Róbert Mészáros, Ádám Leelőssy, Tibor Kovács, István Lagzi

**Affiliations:** 1 Department of Meteorology, Eötvös Loránd University, Budapest, Hungary; 2 Institute of Radiochemistry and Radioecology, University of Pannonia, Veszprém, Hungary; 3 Department of Physics, Budapest University of Technology and Economics, Budapest, Hungary

## Abstract

Long-range simulation of the dispersion of air pollutants in the atmosphere is one of the most challenging tasks in geosciences. Application of precise and fast numerical models in **risk management and decision support** can save human lives and can diminish consequences of an accidental release. Disaster at Fukushima Daiichi nuclear power plant has been the most serious event in the nuclear technology and industry in the recent years. We present and discuss the results of the numerical simulations on dispersion of Fukushima-derived particulate ^131^I and ^137^Cs using a global scale Lagrangian particle model. We compare concentrations and arrival times, using two emission scenarios, with the measured data obtained from 182 monitoring stations located all over the Northern Hemisphere. We also investigate the homogenization of isotopes in the atmosphere. Peak concentrations were predicted with typical accuracy of one order of magnitude showing a general underestimation in the case of ^131^I but not for ^137^Cs. Tropical and Arctic plumes, as well as the early detections in American and European midlatitudes were generally well predicted, however, the later regional-scale mixing could not be captured by the model. Our investigation highlights the importance of the parameterization of free atmospheric turbulence.

The Fukushima Daiichi nuclear disaster has been the second most serious crisis of a nuclear power plant in the human history. The release period lasted over two months emitting a significant amount of radioiodine, radioxenon and radiocaesium as well as other isotopes such as plutonium^1^,^2^. Besides the local to regional scale impacts of soil and water pollution, isotopes released into the atmosphere could be measured globally^3^. Total atmospheric release was estimated to be 14000–15300 PBq of radioxenon and 340–800 PBq of other isotopes^4^. The radioactive plume in the atmosphere moved towards the Pacific, reached North America in five days and Europe in eight days, and returned over Japan around the Northern Hemisphere within only 20 days. Although health concerns have not arisen out of Japan, Fukushima-derived radioiodine and radiocaesium could be identified in the entire Northern Hemisphere as a tracer of atmospheric dispersion. Measurements were made at several sites by international and national monitoring networks as well as research groups and expedition campaigns^3^. The large number of data points allows the identification of transport pathways as well as the evaluation of dispersion model performance and emission scenarios.

While monitoring stations provided valuable data for concentrations in the atmosphere, the emission term and its temporal variability was not known during the accident^5^. The emission was caused by several different processes, including two hydrogen explosions, controlled release and uncontrolled emission for several weeks. Initial estimates of total ^131^I release into the atmosphere ranged from 150 to 500 PBq^1^. Modelling efforts focused on inverse methods that provided more accurate estimates of 65–400 PBq for total ^131^I^1^,^6^. Among others, the WSPEEDI (Worldwide System for Prediction of Environmental Emergency Dose Information) model has been used with data obtained from the Japanese measurement network to estimate a more detailed emission inventory and emission rates, resulting in a total ^131^I emission of 124 PBq and ^137^Cs emission of 8.8 PBq^7^. Recently, the improved WSPEEDI-II model has provided an updated emission data and a detailed reconstruction of the events leading to the accidental release^8^. This latest work concluded a total of 150 PBq ^131^I and 14.4 PBq ^137^Cs emission. Besides the increased total emission compared to the previous estimation, the results of WSPEEDI-II yielded an improved timeline for the first hydrogen explosion and the emission peak on the night 14–15 March^8^.

Here we present a study using a Lagrangian model to evaluate the predictability of the transport of the radioactive plume across the globe comparing modelled concentration values and arrival times to the measured data. We also investigate the improvement of modelled results provided by the WSPEEDI-II emission data compared to earlier reported WSPEEDI emission data. Additionally, based on geographical locations and arrival times we could group the measurement sites into clusters corresponding to different atmospheric transport pathways (Supplementary Figure S1). It should be noted that we focused on dispersion on global scale, therefore, sites within Japan were not investigated in this study.

## Results

### Rapid eastward transport by the jet stream

**Figure 1:**
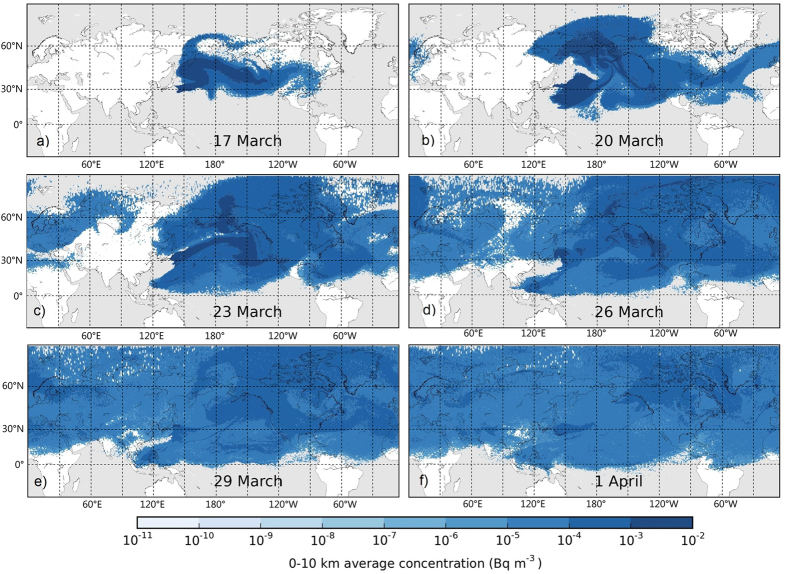
Modelled ^131^I activity concentrations in the troposphere (0–10,000 m) on the 6^th^, 9^th^ 12^th^, 15^th^, 18^th^ and 21^st^ days after the initial release. The figure was made with the Matplotlib Basemap package^60^.

The most important factor in prediction of the global dispersion of Fukushima-derived isotopes was the location of the jet stream. Jet stream is a high tropospheric flow at midlatitudes with mean zonal (westerly) velocities reaching 100 m s^−1^. Cyclones and frontal systems are closely related to meridional (north-south) waves of the jet stream that can reach amplitudes of 1000–3000 kilometres. The pollution cloud was initially captured by an extratropical cyclone over the Western Pacific during the first few days of the release period causing most of the radioactive fallout occurring to the northeast of Japan^3^,^6^,^9^,^10^. The plume was lifted to the middle and high troposphere and, therefore, it was rapidly transported northeastward, arriving to North America only within 5–6 days (Fig. 1 and Supplementary Movie S1)^3^,^4^,^6^,^11^. Strong wind shear in frontal systems over the Eastern Pacific split the plume into two parts: one reaching Alaska and the Canadian Arctic, while the other arriving at the USA West Coast on 16–18 March^3^,^6^,^11^ (Fig. 1a and Supplementary Figure S2). Although the fast transport of pollutants occurred in the strong middle and high tropospheric winds, frontal systems following the jet stream caused efficient vertical mixing that allowed early detection of radioiodine in the planetary boundary layer (PBL)^11^. Vertical turbulence related to frontal systems and deep convection provided efficient vertical mixing in both directions: it was responsible for the initial rapid lifting of the plume as well as the later downward transport to the atmospheric boundary layer (Supplementary Figure S3 and Supplementary Movie S2)^12^.

Arrival of the plume was detected on the same day (17 March) at multiple sites in British Columbia, Washington and California^3^,^11^,^13^. By this time, the plume had reached the high troposphere and was transported eastward in a narrow band by high tropospheric zonal winds reaching velocities of 50–70 m s^−1^ over the Northern Atlantic^3^,^14^ (Fig. 1b and Supplementary Movie S2). Vertical mixing was still provided by frontal convection and precipitation attached to the jet stream, thus Fukushima-derived radioiodine was detected early on the surface following the jet stream pathway in New York State (18 March)^15^, Iceland and Norway (19–20 March)^16^, Sweden, Finland and Western Russia (21–22 March)^17^,^18^ (Supplementary Figures S2 and S3). Over Asia, the jet stream formed meridional waves resulting in a slower and more heterogeneous transport of radioiodine over Asia with arrival to South Korea on 28 March^19^, Taiwan on 29 March^20^ and China only on 31 March^21^. Initial jet stream transport to North America and Europe occurred in the typical height of the jet core (8–10 km) followed by slower and denser low and middle tropospheric plumes. Therefore, concentration peaks occurred several days after the arrival time with values ranging 1.2–14 mBq m^−3^ in North America, 0.6–3.8 mBq m^−3^ in Northern Europe and 0.1–8 mBq m^−3^ in Asia for particulate ^131^I^3^.

### Alaska and the Canadian Arctic

After the initial plume from Fukushima had split into two parts over the Western Pacific, the northern arm reached the Arctic at approximately the same time as the southern arm arrived at the West Coast^11^. The first detection was reported from the Aleutian Islands on 17 March^22^, however, further intrusion to the Arctic was temporarily blocked by a high pressure system. A developing polar cyclone captured the plume and transported northward with arrival times 18–21 March in Alaska and 21–23 March in the Canadian Arctic^3^,^13^,^22^ (Fig. 1c and Supplementary Figure S2). Peak concentrations were in the range of 2.9–25.5 mBq m^−3^ for particulate ^131^I^3^,^13^,^22^. The plume was further transported through the Arctic to Svalbard and Northern Europe^23^,^24^.

### Regional dispersion over North America

The zonal jet stream transported the plume across North America within one day in a narrow band over the Northern USA. This line as well as further Fukushima-derived plumes arriving at the West Coast were the initial state of the regional dispersion dominated by low and middle tropospheric winds. Early detections were reported between 17 and 19 March from three stations in Central and Southern USA: Ashland, KS, Chapel Hill, NC and Melbourne, FL^3^,^22^,^25^. Kansas and North Carolina were reached by a fast moving cold front at the detection time, however, Florida was dominated by a subtropical high pressure system until 21 March. The plume arrived to Orlando, FL, located in a distance of 100 km from Melbourne, FL, only on 25 March with a much lower peak concentration^3^. Other North American stations reported arrival times between 20 and 25 March except those located at the zonal jet stream path. During this period, the homogenization was driven by fast moving low pressure systems with strong horizontal and vertical turbulence as well as precipitation. These effects resulted in fast transport and dilution of the pollutants with arrival times ranging only 5 days but peak concentrations between 0.44 and 31.08 mBq m^−3^ for particulate ^131^I^3^.

### Regional dispersion over Europe

The large number of European measurement sites provided a fine spatial resolution of data from which European dispersion patterns have been identified^17^. The plume was transported to Europe by the zonal jet stream through the North Atlantic and arrived to Scandinavia on 20–22 March (Fig. 1 and Supplementary Figure S2). At this time, a strong high pressure system was blocking Western and Central Europe with sea level pressures exceeding 1030 hPa on a large area from England to Western Russia. Regional transport over Europe occurred within this high pressure system that remained stable until 25–26 March. Following the anticyclonic flow, the first observations and highest peak concentrations were reported in Northern and Eastern Europe with arrival times 21–24 March through Belgium, Germany, Denmark, Belarus, Poland and the Carpathian Basin^17^,^26^,^27^,^28^,^29^,^30^,^31^. By 23–26 March, the plume had been detected in entire Europe except in Southern Italy where a subtropical high pressure system blocked the dispersion until 27–30 March^24^,^27^,^32^,^33^,^34^,^35^,^36^,^37^,^38^,^39^,^40^,^41^,^42^,^43^,^44^,^45^,^46^,^47^,^48^,^49^. During this time, another transport pathway was also playing role from the Pacific through the high Arctic that reached Svalbard on 25 March and merged with zonally transported plumes over Northern Europe^23^,^24^. As regional transport over Europe was dominated by weak anticyclonic winds with no significant precipitation and inefficient vertical mixing, detection times and concentrations were highly dependent on local weather thus showed strong spatial and temporal variability. The homogenization over Europe was slow and heterogeneous with arrival times ranging 10 days and peak concentrations between 0.2 and 9.6 mBq m^−3^ for particulate ^131^I, often with multi-peak concentration time series^3^,^17^,^29^.

### Tropical Central Pacific

Despite their relative vicinity to Japan, the Pacific islands of Guam and Wake Island remained intact for the first week of the accident as the initial plume was captured by an extratropical cyclone and transported northeastward. The Hadley cell was penetrated by frontal systems over the Central and Western Pacific on 16–18 March^3^. Isotopes were transported eastward by trade winds and merged with later emitted plumes penetrating the Hadley cell by multiple frontal zones. Tropical convection provided efficient vertical mixing, thus relatively high surface concentrations (2.3–24.4 mBq m^−3^) were detected with arrival times 19–21 March on Wake Island, Hawaii, Guam and the Northern Mariana Islands^3^,^22^.

### Tropical Western Pacific

Huh *et al*. (2012) pointed out an important transport pathway from Fukushima to Southeastern Asia between 17 and 24 March^20^. With the emission still being significant, a strong frontal system over Japan transported the isotopes in southwestern direction penetrating the Hadley cell over the Western Pacific. The plume was transported in the boundary layer by trade winds and was detected in Manila (23 March)^3^, Taiwan (25 March)^20^, Hongkong and Vietnam (27 March)^50^. Taiwan was also reached by a high tropospheric plume via global jet stream transport on 29 March (Fig. 1e and Supplementary Figure S2)^20^.

### Dispersion over Asia

Due to their vicinity to Japan, measurements were carried out at several sites in China and South Korea. However, no direct eastward transport was detected from Fukushima, and the area was first reached by the high tropospheric plume transported around the globe. The first detection was reported at all 12 South Korean stations on 28 March and at all 24 Chinese stations on 31 March – 1 April^3^,^19^.

A cyclonic system reached South Korea on 28 March causing downward mixing directly from the global jet stream transport. The later detections in China can be explained with a ridge causing mean sea level pressures exceeding 1030 hPa blocking the plume for several days.

### Global arrival times and peak concentrations

**Figure 2:**
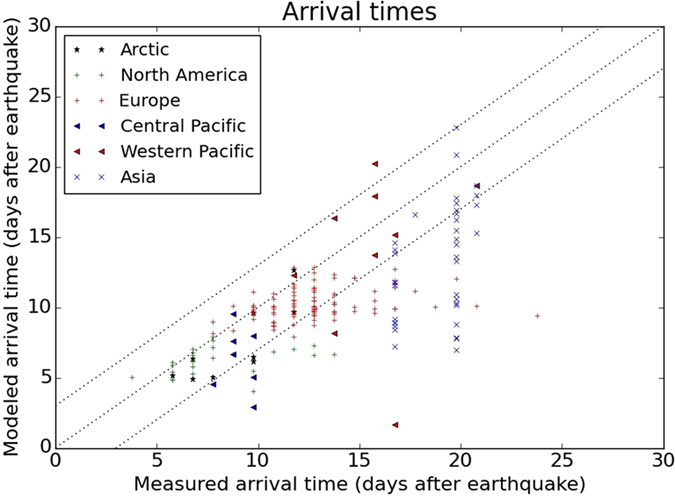
Comparison of measured and modelled arrival times for particulate ^131^I and ^137^Cs.

**Figure 3:**
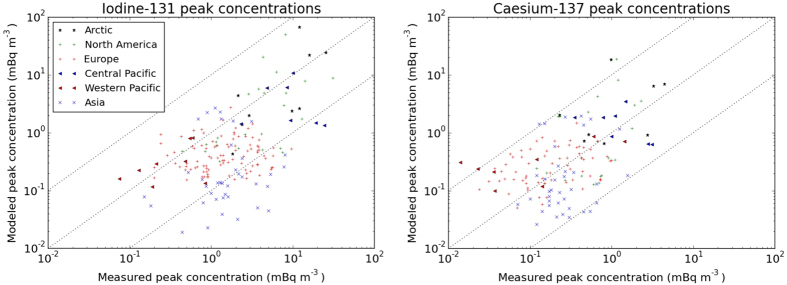
Comparison of measured and modelled peak daily average concentrations for particulate ^131^I and ^137^Cs

Concentrations were simulated with a Lagrangian trajectory model for the first 35 days of the release (Fig. 1 and Supplementary Figure S2). Arrival times representing both iodine and caesium and peak daily concentrations for each isotope are shown in Figures 2 and 3. Intercontinental transport occurred mostly in the middle and high troposphere and main dispersion patterns were well predictable in the free atmosphere. However, vertical mixing from the high troposphere to the planetary boundary layer largely depended on the more uncertain processes of meso-scale turbulence^12^,^51^, frontal zones, convection and precipitation. Planetary boundary layer transport also played a significant role in the Western Pacific^20^. The plume location was mostly well predicted and surface arrival times had an uncertainty of a few days with mean absolute error (MAE) of 2.8 days (Fig. 2). Modelled ^131^I peak concentrations were within the range of one order of magnitude to the measurements (Fig. 3). Values for root mean squared error (RMSE) and coefficient of variation (CV, RMSE divided by the mean of the measured values) at each region are given in Table 1. This level of uncertainty is comparable to that of other model results^6^,^8^,^14^,^52^.

Measurement data points were split into six regions as described previously (Figs 2 and 3). The initial rapid global transport by the jet stream was well predicted by the model with mean absolute error of 2.4 and 2.7 days for arrival times at North American and European stations, respectively. This uncertainty is comparable to the temporal variance of arrival time among stations.

**Figure 4:**
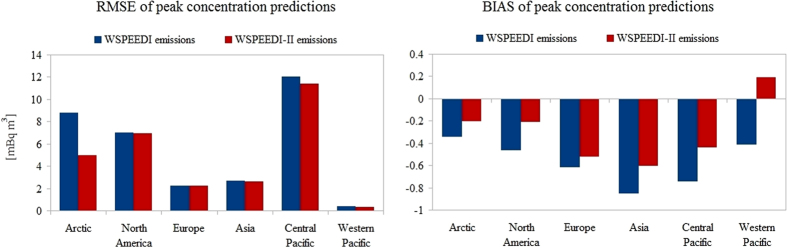
Improvement of global peak particulate ^131^I concentration predictions by updated emission inventory compared to earlier emission estimates^7^,^8^.

Peak concentrations of ^131^I were predicted with similar uncertainty along the zonal transport pathway through North America, Europe and Asia. However, the negative values of the mean relative error (BIAS) showed an overall underestimation of peak ^131^I concentrations (Fig. 4). Peak concentrations of ^137^Cs do not show this general underprediction and have a smaller RMSE, however, the relative error is generally larger.

The large regional variability of peak concentrations within the USA was captured, and model results remained in the same order of magnitude with measurements with the exception of two extremely large peaks in Utah and Indiana. Although the plume arrival times at Arctic stations were similar to those of North America, predictability in the Arctic was better.

In Europe, mixing occurred under anticyclonic conditions. High tropospheric transport of the plume through the Northern Atlantic was captured by the model and early surface detections were well predicted in Iceland and Scandinavia. The general transport and magnitude of the plume were well modelled over Europe, however, the large regional and local variability of concentrations caused worse prediction of the latest arrivals and the largest peaks. In the Tropical Pacific regions, peak concentrations were generally better predicted than those of extratropical latitudes. Arrival times were generally well predicted, however, a few early predictions were related to false locations of westward moving plumes in Southeast Asia.

### Comparison of emission scenarios

The latest emission estimate of Katata *et al*.^8^ (WSPEEDI-II) was compared to their earlier results^7^ (WSPEEDI) to demonstrate the sensitivity of global results on the improved local scale deposition scheme used in WSPEEDI-II. Peak ^131^I concentration results improved in all regions as the larger total emission of WSPEEDI-II reduced the general underestimation of concentrations (Fig. 4). The largest improvement was observable in the Arctic, while other regions showed only a minor change in RMSE but a significantly better BIAS. Similar improvement was not found in case of ^137^Cs, where the increased total amount of the release resulted in a general overestimation of peak concentrations (Supplementary Figure S4).

Large difference between the two emission scenarios was observable in Southeast Asia. Using the WSPEEDI-II emission data, several days of improvement of arrival time predictions was achieved in case of Manila (Philippines), Dalat (Vietnam) and Hongkong (China). This supports the corrected estimation of the 15 March emission by WSPEEDI-II^8^ as this release period corresponded to the boundary layer transport towards Southeast Asia^20^. General accuracy of peak concentrations has also largely improved in this region (CV from 0.92 to 0.76 in case of ^131^I; and from 1.58 to 1.07 in case of ^137^Cs), while the positive BIAS was due to the overprediction of peak concentrations in Vietnam in the WSPEEDI-II scenario.

### Hemispheric homogenization

**Figure 5:**
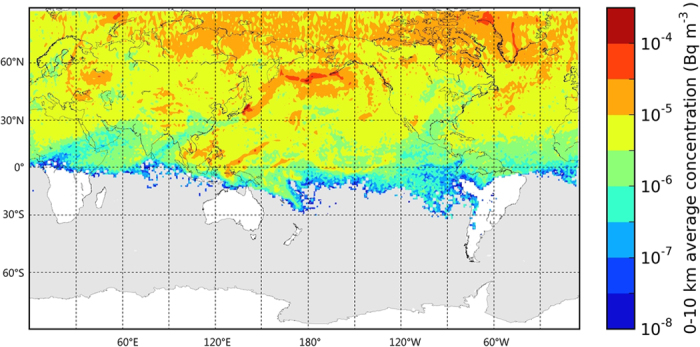
Simulated average tropospheric (0–10 km) particulate ^131^I activity concentration on 11^th^ of April after 31 days of the earthquake. The figure was made with the Matplotlib Basemap package^60^.

**Figure 6:**
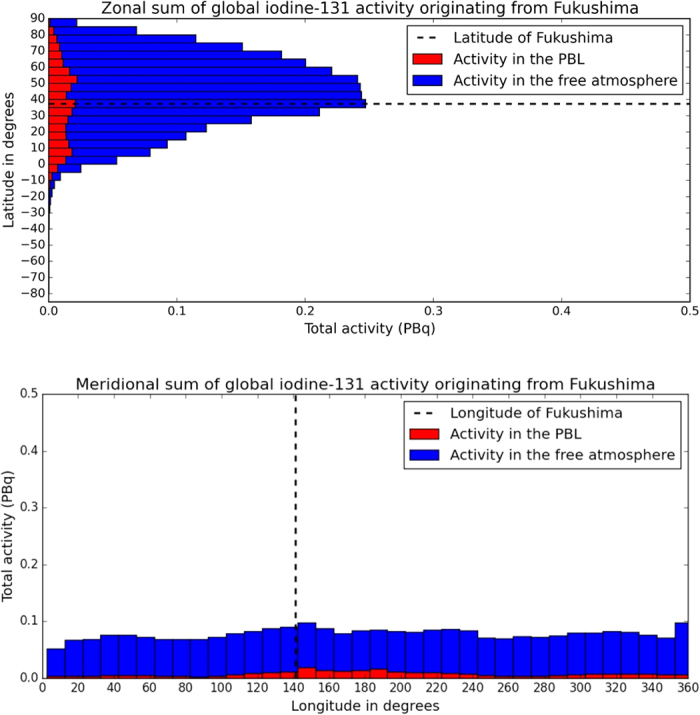
Zonal and meridional homogenization of particulate ^131^I in the atmosphere on 11^th^ of April after 31 days of the earthquake.

The very long duration of the emission from Fukushima was comparable to meridional homogenization time (Fig. 5). Zonal and meridional total activities were integrated one month after the earthquake with 5 degree zonal and 10 degree meridional resolution (Fig. 6). Although hemispheric homogenization did not apply as zonal total activities showed a defined peak at midlatitudes, the region between 40 and 60° N was already well mixed holding 34% of total global atmospheric activity of ^131^I. The tropical regions were dominated by the Hadley cell, a stable and relatively closed circulation pattern that is only rarely penetrated by extratropical cyclones. Southward transport was blocked by tropical high pressure regions and the intertropical convergence zone. Therefore, only 1.3% of the global ^131^I activity was over the Southern Hemisphere at the end of the first month (Figs 5 and 6). A significant intrusion to the southern tropics can be observed towards Australia and the Southwestern Pacific in Fig. 6. After the first month of the dispersion, 16% of the atmospheric ^131^I activity was over tropical latitudes and 20% in northern Polar Regions. The northward gradient of concentration can be explained by the fact that the atmospheric lifetime of ^131^I is shorter in tropical regions due to efficient wet deposition processes.

## Discussion

Predictability of the arrival time of the plume was different among regions, and depended largely on the complexity of the atmospheric circulation systems governing the dispersion. The best correlations can be observed in the tropical and Arctic regions due to the relatively stable trade and polar winds. In American and European midlatitudes, the early detections were generally well predicted by the model, however, the later regional-scale mixing could not be captured, significantly underestimating the arrival times at several sites.

It should be noted that concentration patterns and model performance was studied for a limited number of measurement sites and therefore may not be representative for entire regions. The density of measurement network was fine in Europe, North America and Eastern-Southeastern Asia, however, much coarser in Pacific and Arctic regions. Therefore, measurements in the latter regions are less representative for the absolute peak concentration and predictability of the entire area. The number of measurement sites by region is shown in Table 1.

In Asia, there is a contradiction in the westward moving plumes between 20 and 28 March. Reported measurements in South Korea and China suggest that the first detections were caused by the global hemispheric transport on 28–31 March^3^,^19^,^53^. This arrival time of the high tropospheric plume is supported by our model, however, model results showed earlier surface detections in South Korea between 21 and 26 March and a slow mixing over China between 21 and 31 March due to westward transport from Fukushima (Fig. 1 and Supplementary Fig. 2). It has been shown in previous model simulations that an anticyclonic system forced the plume to northwestward direction between 20 and 24 March that could marginally reach Northeastern China and the Korean Peninsula^3^,^6^,^20^. Our model also suggests further westward dispersion to mainland China by 26 March. However, this early transport was not supported by other model and measurement results^3^,^14^,^19^,^53^. Contrary to these, detection of Fukushima-derived radionuclides was reported from a study in Xi’an, Central-Eastern China on 23 March with a significant peak on 25 March^54^, more according to our estimates. To assess this contradiction, we also used results from the online version of HYSPLIT (Hybrid Single Particle Lagrangian Integrated Trajectory Model) applied for the Fukushima case using the Transfer Coefficient Matrix (TCM) method^52^. The concentration timeline for Xi’an and Seoul provided by HYSPLIT radically supported the scenario of a significant westward transport with arrival times as early as 15–16 March in Xi’an and Seoul. HYSPLIT also showed the reported peak in Xi’an on 25 March. The high uncertainty of the pathway and surface detectability of early westward moving plumes from Fukushima points out the importance of further studies.

The initial ratio of gaseous to total ^131^I was obtained from the emission scenario^8^, however, this ratio was decreased by wet scavenging of aerosols throughout the dispersion. Wet scavenging of ^131^I is one of the most uncertain processes of atmospheric dispersion that is highly dependent on the ratio of the chemical forms of iodine in the plume as well as precipitation and cloud data obtained from global meteorological models. Despite neglecting all sink processes except radioactive decay and wet scavenging, our results showed an overall underprediction of ^131^I peak concentrations, a similar phenomena that had been assessed in previous modelling studies^14^,^52^.

**Figure 7:**
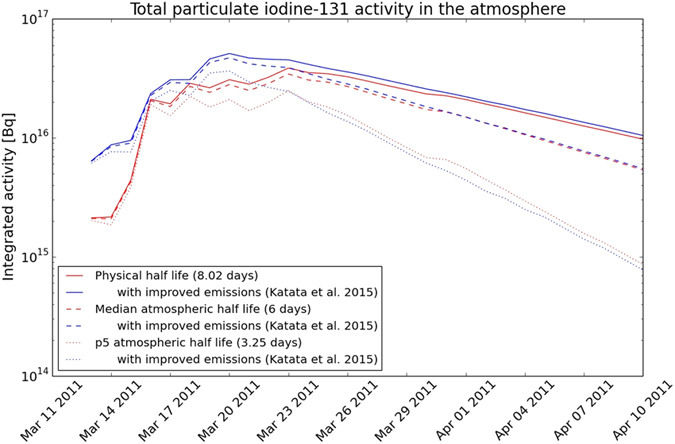
Comparison of total atmospheric activity of particulate ^131^I with initial^7^and improved^8^ emission scenarios. Decay rates among the physical half-life of 8 days and reported median and 5% percentile (p5) atmospheric half life of 6 and 3.25 days^29^, respectively, are compared.

Initial gaseous/total ratio was measured between 30 and 67% in Japan^55^ and 40 and 100% in Europe and North America^3^. Wet scavenging was shown to decrease the contribution of particle form to the total, therefore largest gaseous/total ratios were measured at locations where transport was permanently accompanied with precipitation, mostly related to the jet stream^3^. The development of WSPEEDI-II for the latest emission estimate focused largely on the representation of different deposition processes for gaseous and particulate iodine^8^. They assumed 50% gaseous/total ratio for the initial, largest emissions and an integrated ratio of 54% for the entire release^8^. Based on measurements in North America and Europe, an average atmospheric ratio of 76.7 ± 12% was calculated for the period until 22 April^3^. This yields an average uncertainty of concentrations with a factor of 1.5 due to wet scavenging which is comparable to the uncertainty of the initial ratio of aerosol and gas forms. Atmospheric half-life of particulate ^131^I was estimated to be 6 days (median) with a range between 3.25 and 8.5 days^28^,^29^. Figure 7 clearly shows that the uncertainties caused by deposition processes dominate over uncertainties of emission scenarios only after 20–25 March.

It has been shown previously that in atmospheric dispersion problems, exchange of pollutants between the planetary boundary layer and the free atmosphere plays a significant role^56^. On regional to global scales, downward convective vertical transport can contribute significantly to surface concentrations^12^. These effects are often poorly represented in dispersion models. In this study, we applied constant diffusion coefficients above the planetary boundary layer. However, we performed model runs using various scenarios for turbulent mixing in the free atmosphere, and our study revealed that improper parameterization of the free atmospheric turbulence give rise to errors greater than one order of magnitude in the prediction of surface concentrations. Parameterization of turbulent processes in the free atmosphere would provide new directions for further studies in air quality modelling.

## Methods

In this study we used the RAPTOR dispersion model for the simulation of arrival times and ^131^I activity concentrations in the atmosphere for a 35-day period. This continental/global scale trajectory model was developed at the Eötvös Loránd University, Hungary. Four million particles were emitted and their trajectories were calculated for the entire simulation time. Each particle represented a fraction of the total activity that decreased with radioactive decay and wet deposition over time. The model used longitude–latitude horizontal and *z* (absolute) vertical coordinates. Digital elevation data from the Global Land One-km Base Elevation Project^57^ was used to provide more accurate results of plumes traversing complex terrain. Particles were moved along air parcel trajectories determined by the sum of the grid-scale wind and the subgrid-scale stochastic turbulent velocity, solving the ordinary differential equation with a simple first-order forward scheme. The turbulent velocities were calculated using the Langevin equation with turbulent parameters obtained from Hanna’s parameterization^51^,^58^. Atmospheric stability was characterized with the Monin–Obukhov-length calculated directly from heat flux data provided by the meteorological model. Aerosol wet scavenging was parameterized with a simple first-order deposition scheme assuming the same size distribution for both iodine and caesium aerosols. Scavenging coefficients were calculated separately from large-scale and convective precipitation rates using empirical parameters given by Leadbetter *et al*. (2015)^59^.

To save computational time, only the two major sink processes: radioactive decay and wet scavenging were taken into account. The incorporation of surface dry deposition requires land use data and surface-atmosphere interaction parameterizations that largely increase the computational cost and the amount of data involved. This increase in computational cost is not proportionate to the improvement of concentration results, having uncertainty of one order of magnitude compared to measurements. One could use a constant dry deposition velocity, however, this would not take into account the spatial and temporal variability of dry deposition (as it is taken into account in the case of wet scavenging through precipitation intensity). It is more justifiable to introduce a slight general underestimation by neglecting the dry deposition than a slight bias in time- and space-dependent direction by defining a constant dry deposition velocity.

Input meteorological data was obtained from the Global Forecast System (GFS, http://www.emc.ncep.noaa.gov) with horizontal resolution of 0.5 degree. To maximize meteorological data accuracy with keeping the 3 hourly temporal resolution, we used the 3 and 6 hour forecast files of consecutive GFS runs. Three-dimensional fields and model vertical velocities were interpolated from pressure to absolute height levels. GFS provides 3D wind field for the trajectory simulation, while 2D meteorological data (height of the planetary boundary layer, heat fluxes, surface wind speed and temperature) were used to estimate the Monin–Obukhov-length and the turbulent velocities. Concentrations were calculated from trajectory positions every hour on the grid of the input meteorological data with 0.5 degree resolution. Model concentration outputs were averaged for 24 hours for comparison with measurement data from 24-hour samples^29^. The model was implemented in the Anaconda Python framework^60^.

Measurement data was mostly obtained from the review of Thakur *et al*.^3^ who published data for several CTBTO (Comprehensive Nuclear-Test-Ban Treaty Organization) sites as well as observations from national networks and research institutions. Besides referenced publications and the CTBTO, original data sources had been the US Environmental Protection Agency, the University of California, the Washington State Department of Health, national radiation safety authorities and public research centres of European countries and the Ministry of Environmental Protection of China. Emission data (emission rate and amount) was obtained from Katata *et al*.^8^ and their earlier work^7^.

## Additional Information

**How to cite this article**: Mészáros, R. *et al*. Predictability of the dispersion of Fukushima-derived radionuclides and their homogenization in the atmosphere. *Sci. Rep.* **6**, 19915; doi: 10.1038/srep19915 (2016).

## Supplementary Material

Supplementary Movie 1

Supplementary Movie 2

Supplementary Information

## Figures and Tables

**Table 1:** Comparison of modelled peak concentrations to measurements in each region.

Region	Number of measurement sites (^131^I/^137^Cs)	Aerosol^131^I			Aerosol^137^Cs		
		Measured mean concentration (mBq m^−3^)	RMSE(mBq m^−3^)	CV	Measured mean concentration (mBq m^−3^)	RMSE (mBq m^−3^)	CV
Arctic	8/8	9.4	4.9	0.5	1.7	1.8	1.1
North America	23/22	5.8	6.9	1.2	0.77	1.9	2.5
Europe	95/64	2.0	2.2	1.1	0.26	0.31	1.2
Asia	37/37	2.1	2.6	1.3	0.33	0.62	1.9
Central Pacific	7/7	11.2	11.4	1.0	1.5	1.6	1.1
Western Pacific	8/8	0.38	0.29	0.76	0.30	0.32	1.1
