# Supplementary material for: Predictability of the dispersion of Fukushima-derived radionuclides and their homogenization in the atmosphere
Source: Sci Rep. 2016 Jan 28;6:19915. doi: 10.1038/srep19915 (PMC4730138; doi:10.1038/srep19915)
Supplement: Supplementary Information [file srep19915-s3.pdf]

SUPPLEMENTARY INFORMATION

SUBJECT AREAS ENVIRONEMTAL SCIENCES

Correspondence and requests for materials should be addressed to R.M. (mrobi@nimbus.elte.hu)

Predictability of the dispersion of Fukushima-derived radionuclides and their homogenization in the atmosphere

Róbert Mészáros[1], Ádám Leelőssy[1], Tibor Kovács[2], István Lagzi[3]

Affiliations

[1]Department of Meteorology, Eötvös Loránd University, Budapest, Hungary

[2]Institute of Radiochemistry and Radioecology, University of Pannonia, Veszprém, Hungary

[3]Department of Physics, Budapest University of Technology and Economics, Budapest, Hungary

Supplementary Figures

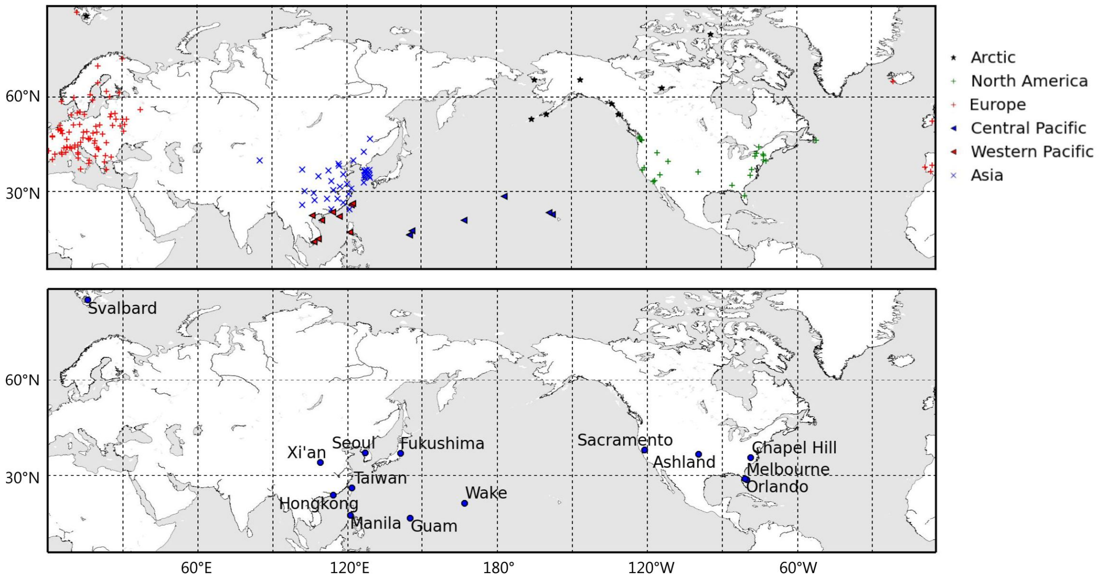

Supplementary Figure S1. Location and classification of measurement sites (up) and map of a few locations explicitly mentioned in the paper (down). The figure was made with the Matplotlib Basemap package.[1]

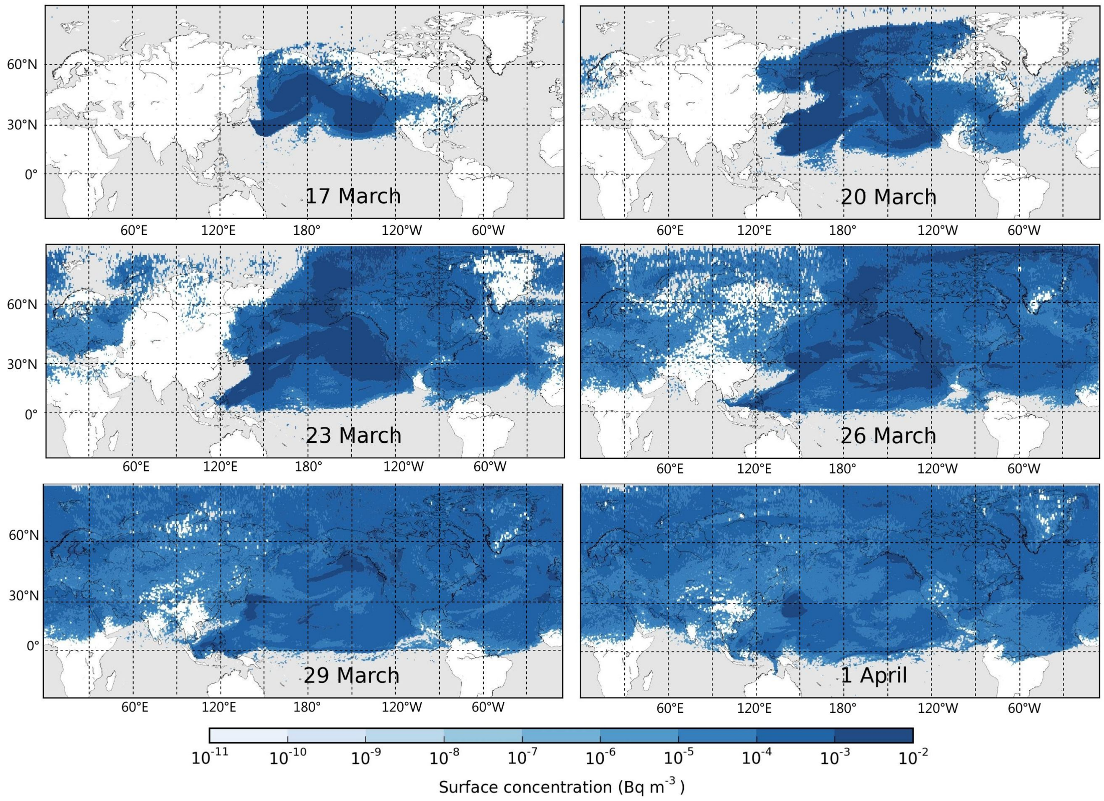

Supplementary Figure S2. Modelled ^{131}I activity concentrations in the surface layer (0 – 100 m) on 6th, 9th 12th, 15th, 18th and 21st days after the initial release. The figure was made with the Matplotlib Basemap package.[1]

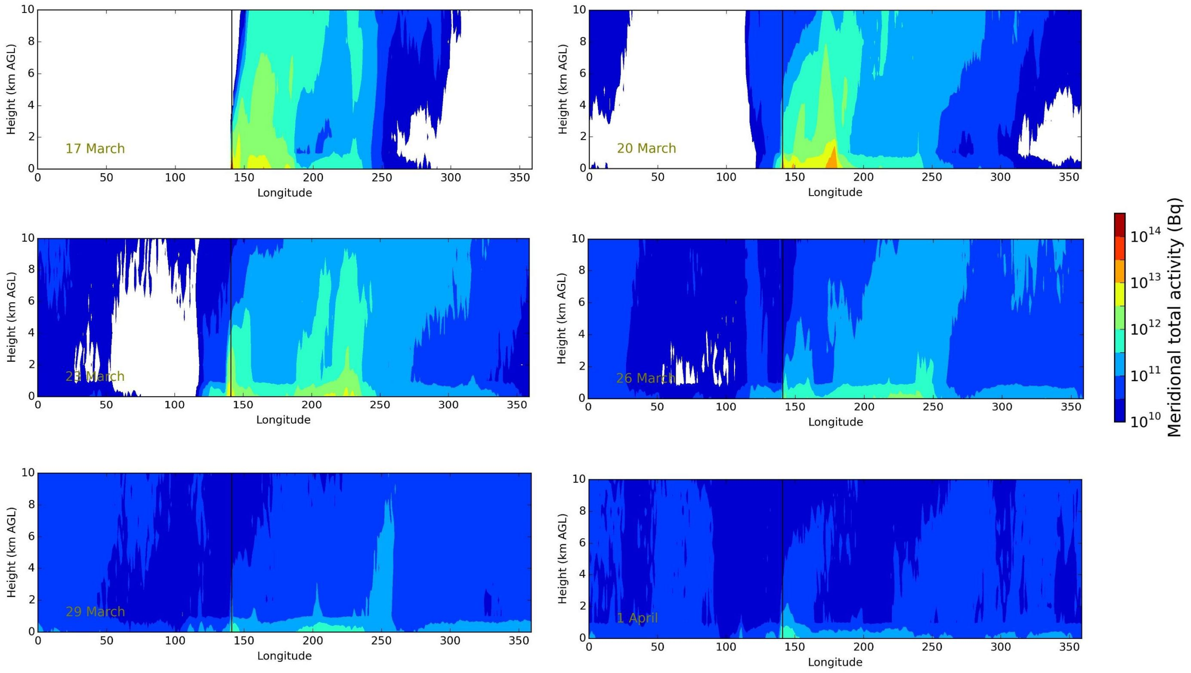

Supplementary Figure S3. Vertical profile of meridionally integrated ^{131}I activity in the troposphere (0 – 10,000 m) on 6th, 9th 12th, 15th, 18th and 21st days after the initial release. The figure was made with the Matplotlib package.[1]

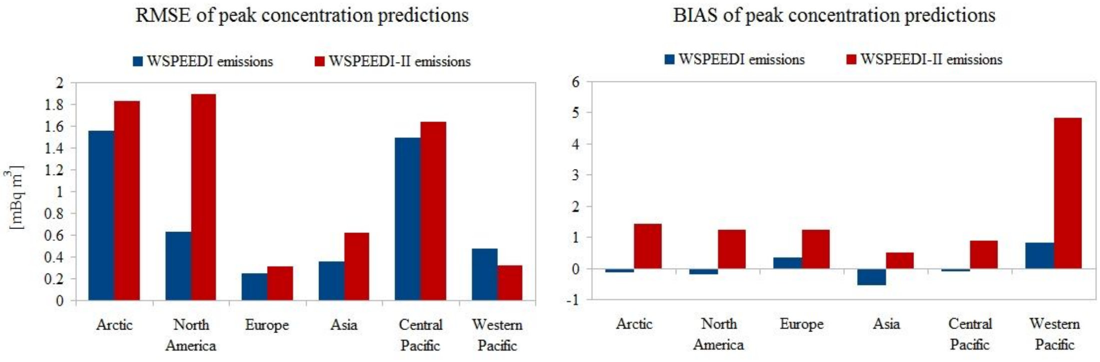

Supplementary Figure S4. Comparison of peak concentration results for particulate ^{137}Cs concentration between WSPEEDI and updated WSPEEDI-II emission scenario.

Supplementary Movies

Supplementary Movie S1

Modelled ^{131}I activity concentrations in the surface layer (0–100 m) on the 6th, 9th 12th, 15th, 18th and 21st days after the initial release. The figure was made with the Matplotlib Basemap package.[1]

Supplementary Movie S2

Modelled vertical distribution of meridionally integrated particulate ^{131}I activity in the atmosphere on the 6th, 9th 12th, 15th, 18th and 21st days after the initial release. The figure was made with the Matplotlib package.[1]

References

1. Hunter, J. D. Matplotlib: A 2D Graphics Environment. *Computing in Science Engineering* **9,** 90–95 (2007).
